# Multidisciplinary Treatment for Lymphorrhea and Chylorrhea Following Lymph Node Dissection for Genitourinary Cancer

**DOI:** 10.3390/cancers17040592

**Published:** 2025-02-10

**Authors:** Naoto Kamiya, Takahide Noro, Taro Okazaki, Naoki Ishitsuka, Yuta Suzuki, Shota Iijima, Yuka Sugizaki, Takatoshi Somoto, Ryo Oka, Takanobu Utsumi, Takumi Endo, Shusuke Kasuya, Nobuyuki Hiruta, Hiroyoshi Suzuki

**Affiliations:** 1 Department of Urology, Toho University Sakura Medical Center, 564-1 Shimoshizu, Sakura-shi 285-8741, Chiba, Japan; takahide.noro@med.toho-u.ac.jp (T.N.); taro.okazaki@med.toho-u.ac.jp (T.O.); naoki.ishitsuka@med.toho-u.ac.jp (N.I.); yuta.suzuki@med.toho-u.ac.jp (Y.S.); shouta.iijima@med.toho-u.ac.jp (S.I.); yuuka.kizuki@med.toho-u.ac.jp (Y.S.); takatoshi.soumoto@med.toho-u.ac.jp (T.S.); ryou.oka@med.toho-u.ac.jp (R.O.); takanobu.utsumi@med.toho-u.ac.jp (T.U.); takumi.endou@med.toho-u.ac.jp (T.E.); hiroyoshi.suzuki@med.toho-u.ac.jp (H.S.); 2 Department of Radiology, Toho University Sakura Medical Center, 564-1 Shimoshizu, Sakura-shi 285-8741, Chiba, Japan; kasuya-shu@sakura.med.toho-u.ac.jp; 3 Department of Surgical Pathology, Toho University Sakura Medical Center, 564-1 Shimoshizu, Sakura-shi 285-8741, Chiba, Japan; nhr@med.toho-u.ac.jp

**Keywords:** lymphorrhea, chylorrhea, lymph node dissection, octreotide, interventional radiology, intranodal lymphangiography, lymphatic embolization

## Abstract

Background: Lymph node dissection (LND) is often performed in genitourinary cancer to improve accurate staging. However, the resultant lymphatic damage often leads to postoperative lymphorrhea and chylorrhea. Further, since lymphatic fluid lacks platelets, it has very few clotting factors, and it is often difficult to treat postoperative lymphatic leakage. Treatments for lymphorrhea include conservative treatment (e.g., fasting, total parenteral nutrition, and drug therapy), interventional radiology (IR) and surgical treatment. However, there is no guideline of refractory lymphorrhea, and no clear criteria for switching to the next treatment. Methods: We reviewed the records of 28 patients at Toho University Sakura Medical Center with postoperative lymphorrhea or chylorrhea after LND that did not improve with conservative treatment. Based on this analysis, we partially revised the treatment algorithm for lymphorrhea developed by Rose et al. Results: The cases consisted of 26 men and two women, aged 65.0 ± 9.9 years. The mean number of lymph nodes removed was 25.3 ± 15.0. Octreotide was administered in 27 patients, lymphangiography was performed in three patients, and lymphatic embolization was performed in two patients. The mean duration of octreotide administration was 9.7 ± 6.1 days, and the mean dose was 211.1 µg/day. The treatment success rates with octreotide and IR were 78.6% and 100%, respectively. The mean duration of drain placement after surgery for primary cancer was 18.3 ± 14.3 days. Conclusions: Patients with lymphorrhea and chylorrhea should be initially treated conservatively, with IR performed if conservative treatment is unsuccessful. Surgical treatment should be a last resort.

## Simple Summary

Lymph node dissection (LND) damages the lymphatic pathway, leading to postoperative lymphorrhea and chylorrhea. Treatments for lymphorrhea include conservative treatment (e.g., fasting, total parenteral nutrition), drug therapy, interventional radiology (IR), and surgery. We reviewed the records of 28 patients at Toho University Sakura Medical Center with postoperative lymphorrhea or chylorrhea after LND that did not improve with conservative treatment. Based on our analysis, we partially revised the treatment algorithm for lymphorrhea developed by Rose et al. Octreotide was administered in twenty-seven patients, lymphangiography was performed in three patients, and lymphatic embolization was performed in one patient. Treatment success rates for octreotide and IR were 78.6% and 100%, respectively. The mean duration of drain placement after surgery for primary cancer was 18.3 ± 14.3 days. Patients with lymphorrhea and chylorrhea should be initially treated conservatively, with IR performed if conservative treatment is unsuccessful. Surgical treatment should be a last resort.

## 1. Introduction

Lymph node dissection (LND) in genitourinary cancer is performed for the purpose of improving prognosis and performing accurate cancer staging [1,2,3,4]. However, it can lead to accidental damage to lymph vessels and cause iatrogenic lymph leakage. Many types of postoperative lymph leakage have been reported, including lymphoascites, lymphocele, lymphorrhea, lymphatic fistula, and special forms of lymph leakage, such as chylous ascites, chylous leakage, and chylous pleural effusion [5]. The treatment for postoperative lymphorrhea is conservative or surgical [6]. Postoperative lymphorrhea generally improves with conservative treatment, and only rarely becomes refractory. However, compared to arteries and veins, lymphatic vessels have extremely thin walls and few collagen fibers, and are not sealed by blood coagulation. This is because, in addition to the absence of platelets in lymph, the concentration of coagulation factors is extremely low, making it difficult for lymphatic vessels to naturally repair themselves [7]. Once postoperative lymphorrhea becomes refractory, it can become serious. Intractable lymphorrhea and chylorrhea can be complicated by malnutrition and infection, which can last for several weeks to several months before complete recovery, prolonging the duration of hospitalization and increasing medical costs. Additionally, patients with malignant tumors are faced with the problem of delay in the implementation of adjuvant therapy. In recent years, intranodal lymphangiography (IL) has become possible, and interventional radiology (IR) therapy for postoperative lymphorrhea is rapidly becoming more widespread for the treatment of refractory lymphorrhea [8,9]. There is only one treatment algorithm for chylorrhea after surgery for genitourinary cancer developed by Rose et al., However, there is no guideline for refractory lymphorrhea, and when there is no response to conservative treatment in a short period of time, there are no clear criteria for switching to the next treatment and which the treatment to be selected. Here, we retrospectively analyzed patients with lymphorrhea and chyle leakage that occurred after LND for genitourinary cancer who underwent treatment intervention at our institution. Based on the results of this analysis, we partially revised the treatment algorithm for chylorrhea developed by Rose et al. to shorten the duration of hospital stay and the treatment period for lymphorrhea and chylorrhea [6].

## 2. Materials and Methods

**Figure 1:**
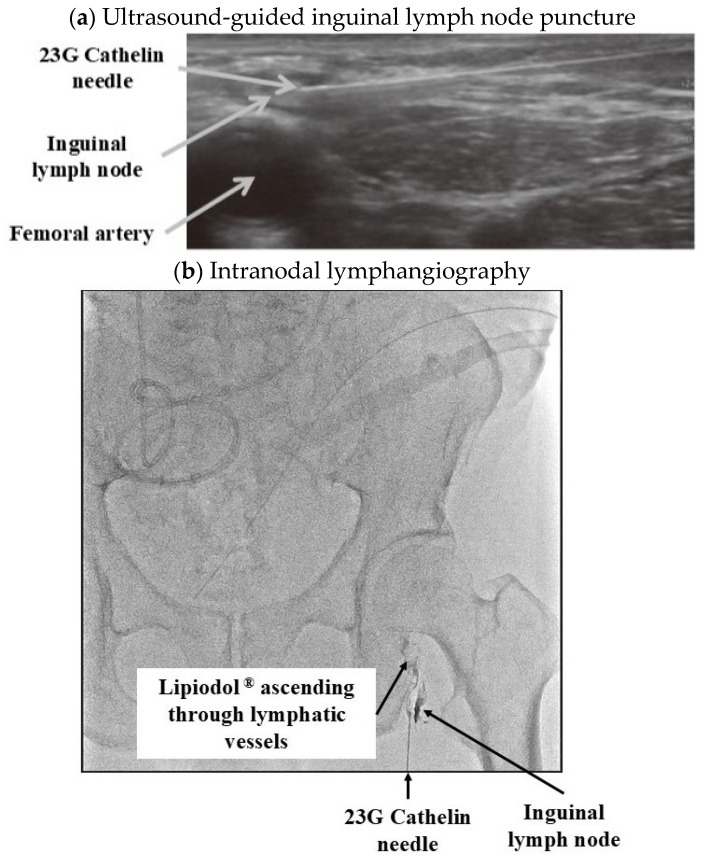
Intranodal lymphangiography for postoperative lymphorrhea.

This retrospective study was conducted according to the principles of the Declaration of Helsinki and was approved by the ethics committee of Toho University Sakura Medical Center (approval number: S24001, approval date: 5 September 2024). We reviewed the records of 28 patients with postoperative lymphorrhea (chylorrhea: four patients) after LND at our institution between September 2014 and March 2024. Medical records were surveyed retrospectively, and the following data were collected from medical charts: age at surgery, sex, primary tumor location, surgical method, approach method, number of LNs removed, N stage (clinical and pathological), number of positive lymph node metastasis, type of leakage, drainage volume (before and after treatment), and duration of drain placement. Although all 28 cases underwent dietary therapy, the study subjects were cases in which the disease could not be cured with dietary therapy alone. In patients with bladder and prostate cancer, either extended pelvic LND, which includes dissection of bilateral common, external, and internal iliac + obturator (+ pre sacral) lymph nodes, or limited dissection of bilateral obturator lymph nodes was performed. LND for renal pelvic cancer included dissection of the renal hilar lymph nodes. Drains were placed in all cases in which lymph node dissection was performed. At our institution, intervention for lymphorrhea or chylorrhea is considered when the amount of drainage fluid is 200 mL/day or more for LND performed by a retroperitoneal approach, and 400 mL/day or more with transperitoneal LND. First, conservative treatment (including dietary therapy, such as fasting or a low-fat diet, and drug therapy, such as octreotide, used in combination) is performed for one week. Total parenteral nutrition (TPN) is performed if the fasting period is prolonged. Drug therapy consists of subcutaneous injections of octreotide (200–300 µg/day). If the drainage volume does not improve after one week of conservative treatment, IR is performed. IR essentially involves IL using Lipiodol^®^ (Guerbet Japan Co., Ltd., Tokyo, Japan), with the decision of whether to perform embolization using N-butyl-2-cyanoacrylate (NBCA) being at the discretion of the radiologist during surgery. Figure 1 shows the IL procedure in one of our cases.

First, the inguinal LN in the groin is punctured freehand using a superficial linear high-frequency probe. Next, the lymph node is percutaneously punctured by connecting an extension tube with a locking syringe to a 23G Cathelin needle. After puncturing the LN, Lipiodol^®^ (Guerbet Japan Co., Ltd., Tokyo, Japan) is slowly injected until bead-shaped lymph vessels are visualized. If there is no improvement in the drainage volume one week after IR, IR is reattempted. Before IR became available, in cases treated using the retroperitoneal approach, where there was no concern about migration to the peritoneum, 200 mg of minocycline was dissolved in 40 mL of saline and injected into the retroperitoneal cavity through the drain as adhesion therapy. If the drainage volume still did not improve, surgical treatment was considered as a last resort.

In this study, we defined the treatment as being successful in cases in which the drain could be removed. In all cases, pathological evaluation of the surgical specimens was performed by two out of three pathologists, as a council system, at Toho University Sakura Medical Center. The results are presented as the median and range or mean ± standard deviation, as appropriate. All statistical analyses were performed using SPSS version 11.0 (SPSS Inc., Chicago, IL, USA).

## 3. Results

Table 1 shows the characteristics of patients who developed postoperative lymphorrhea or chylorrhea.

The mean patient age was 65.0 ± 9.9 years, and males represented 92.9% of the study population. The primary sites of genitourinary cancer were bladder cancer in 14 cases (50%), prostate cancer in 12 cases (42.9%), and renal pelvic cancer in 2 cases (7.1%). The surgical methods for primary cancer and LND were open surgery in 17 cases (60.7%), laparoscopic surgery in 6 cases (21.4%), and robotic-assisted surgery in 5 cases (17.9%). Surgery was performed by the transperitoneal approach in 17 cases (60.7%) and retroperitoneal approach in 11 cases (39.4%). There was no significant difference in the amount of drainage fluid before treatment based on the approach method (transperitoneal: 396.5 ± 508.8 mL, retroperitoneal: 337.4 ± 218.7 mL; N.S.). LND was performed in all cases, and the mean number of lymph nodes dissected was 25.3 ± 15.0. Among them, the mean number of lymph nodes that were positive for metastases was 0.9 ± 3.4. The type of leakage was lymphorrhea in 24 cases (85.7%) and chylorrhea in 4 cases (14.3%). In the overall cohort, the mean daily drainage volume before treatment intervention was 373.3 ± 444.7 mL/day, and the mean daily drainage volume before drain removal (after completion of treatment for lymphorrhea or chylorrhea) was 81.1 ± 128.8 mL/day. The mean duration of drain placement after surgery for primary cancer was 18.3 ± 14.3 days.

**Figure 2:**
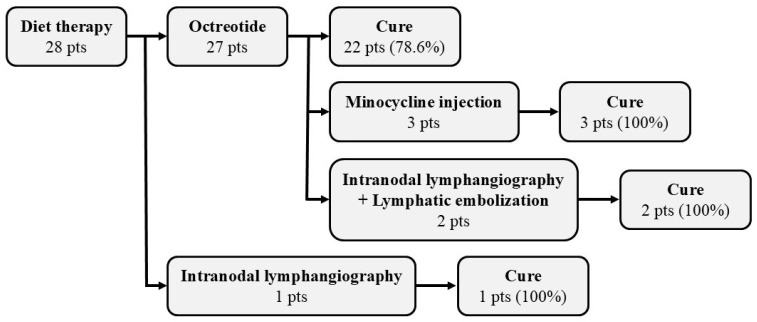
Treatment methods for postoperative lymphorrhea and chylorrhea. pts: patients.

Figure 2 shows the treatment methods and therapeutic effects in lymphorrhea and chylorrhea cases.

Dietary therapy was administered in all cases, and octreotide was used in 27 patients (96.4%). The mean dose of octreotide was 211.1 ± 42.4 mg/day, and the mean duration of administration was 9.7 ± 6.1 mg/day. Twenty-two cases (78.6%) were cured by drug therapy with octreotide. The mean duration of drain placement after surgery for primary cancer was 18.3 ± 14.3 days. None of the patients developed adverse events (AEs) in association with octreotide therapy. Injection of minocycline into the drain for adhesion therapy was performed in three cases (10.7%), resulting in a cure in all of them. IL was performed by a radiologist in three patients (10.7%) in whom lymphorrhea and chylorrhea did not improve with conservative treatment. Since two of these cases had severe leakage, lymphatic embolization using NBCA was additionally performed. Further, since the lymphorrhea or chylorrhea in all patients treated with IL and lymphatic embolization using NBCA was successful, none of the cases at our institution required surgical treatment. There were no cases of AEs associated with IR.

## 4. Discussion

Lymphatic circulation serves to drain proteins and excess interstitial fluid back to the systemic circulation; it regulates immune responses by both cellular and humoral mechanisms, and absorbs lipids from the intestine [7]. After their absorption from the intestine, fatty acids with fewer than 10 carbon atoms are transported directly to the portal system, whereas fatty acids with more than 10 carbon atoms are absorbed into the chyle and lymphatic capillaries of the small intestine to form chylomicrons [10,11]. The mixture of lymph and chylomicrons is called chyle, which is milky white, odorless, and has a strong bactericidal effect due to the high number of lymphocytes [12,13]. During the process of digestion and absorption, 3 to 5 L/day (60 to 200 mL/h) of lymph passes through the thoracic duct [7]. Fasting before surgery dramatically reduces lymph flow to less than 1 mL/min, and returning to a normal diet increases lymph flow to more than 200 mL/min [14].

Before diagnosing lymphorrhea, it is necessary to first rule out other postoperative complications, such as malignant ascites, intraperitoneal hemorrhage, urinary ascites due to bladder injury, malignant pleural effusion, inflammatory exudate in the body cavity, and purulent exudate [15]. Lymphorrhea is often asymptomatic. However, after the drain is removed, abdominal distension, lower abdominal pain, back pain, nausea, difficulty breathing, weight gain, etc., might be observed. Prolonged continuous chyle leakage leads to the leakage of proteins, lymphocytes, and immunoglobulins, resulting in malnutrition and infections associated with immune dysfunction [16].

The incidence of lymphatic fistulas and chyle fistulas following abdominal surgery varies depending on the surgical procedure. In rectal cancer surgery, the incidence rate is reported to be 6.9% for robotic surgery, 4.2% for laparoscopic surgery, and 1.0% for open surgery [17]. In order to prevent postoperative lymphorrhea and chyle leakage, it is important to securely ligate and clip the lymphatic vessels through careful surgical manipulation during surgery. Intraoperative damage to the lymphatic system can cause postoperative lymph leakage. Monopolar, bipolar and ultrasonic coagulation in surgery, in particular, are common causes of damage to the lymphatic tracts [18]. Lymphatic leakage might be missed during laparoscopic surgery due to the effect of the positive CO pressure on the lymphatics, increasing the risk of unrecognized lymphatic vessel injury during surgery [19]. Most lymph node dissections result in the leakage of lymph vessels, which usually resolves spontaneously without causing symptoms of lymphocytic ascites. Lymphatic fistulas typically form after 48 h of continuous leakage [12]. Damage to the lymphatic vessels from the superior mesenteric artery lymphatic system to the intestinal lymphatic trunk, cisterna chyli, and thoracic duct results in chyle leakage, but lymphatic leakage from the celiac artery system, hepatic lymphatic system, and lumbar lymphatic trunk system is less likely to result in chyle leakage [20]. Chyle is an odorless, milky fluid composed of lymph fluid and chylomicrons. Chyle leakage is diagnosed by the presence of milky white drainage from the drain tube after the start of oral intake postoperatively, and by the triglyceride level in the drainage being 110 mg/dl or higher due to the naturally high triglyceride levels in chyle [7,21]. The symptoms of chyle leakage usually occur at an average of 4.1 days after surgery due to the commencement of dietary intake on postoperative day 2. In rare cases, symptoms might occur several months after surgery [22]. Approximately 80% of postoperative abdominal chyle leakages occur following abdominal aortic surgery, although the incidence rate has been reported to be lower than 1% [7]. In the field of urology, chyle leakage has been reported following radical nephrectomy for renal cancer, donor nephrectomy, total nephroureterectomy for renal pelvic or ureteral cancer, and para-aortic lymphadenectomy for testicular cancer. Approximately 1.2% of para-aortic LNDs for testicular cancer are associated with chylorrhea, and preoperative chemotherapy and inferior vena cava resection are considered risk factors for postoperative chylorrhea [22,23]. There are three mechanisms for the formation of chylorrhea: ① direct leakage of chyle through lymphoperitoneal fistulas associated with abnormal retroperitoneal lymphatics; ② seepage of chyle through the walls of retroperitoneal lymphatics without visible fistulas; and ③ seepage or leakage of chyle after rupture of dilated lymphatics in the intestinal wall and mesentery caused by lymphatic obstruction at the mesentery, cisterna chyli, or base of the thoracic duct [24].

Table 2 shows treatments for lymphatic fistulas and chyle fistulas. Lymphatic fistulas and chyle fistulas often improve with conservative treatment alone. In the past, there were occasional reports of surgical interventions, such as ligation of the leakage site or application of fibrin glue, for cases resistant to conservative treatment. However, surgical interventions are highly invasive, and the leakage site might not be identifiable even with surgery [6]. The aim of dietary intervention in the management of lymphorrhea and chylorrhea is to minimize intestinal lymphatic flow and it is the first step in the treatment of patients with lymphorrhea and chylorrhea. During the processes of digestion and absorption in the intestine, long-chain triglycerides are converted to monoglycerides and free fatty acids and transported via the intestinal lymphatics to the cisterna chyli, exacerbating chyle leakage. On the other hand, medium-chain triglycerides (MCTs) are transported via the portal vein as free fatty acids and glycerol, bypassing the intestinal lymphatic circulation. Therefore, dietary treatment with a low-fat diet is more effective when supplemented with MCTs [24]. Depending on the daily lymphatic and chyle output, fasting might be necessary. Total parenteral nutrition is necessary when malnutrition persists for a long time. Although the recommended duration of dietary treatment is not clear, a low-fat diet is usually continued for several weeks after surgery.

Somatostatin was isolated and identified by Brazeau et al. in 1973 and reported as a peptide that inhibits growth hormone secretion [25]. Octreotide, a somatostatin analog, dramatically reduces lymphorrhea within 24 to 72 h after its administration and is an effective treatment with few adverse events. Octreotide acts against chyle leakage by ① reducing thoracic duct flow by suppressing digestive juice secretion due to its gastrointestinal hormone secretion suppressing effect, ② inducing the contraction of smooth muscles via somatostatin receptors present in lymphatic endothelium or smooth muscle, and ③ ultimately reducing portal venous pressure and enterohepatic lymph flow, thus reducing chyle leakage [26,27]. Octreotide has a longer half-life than somatostatin and can be administered subcutaneously. The initial dose is 100 mg three times a day, which can be increased to 200 mg three times a day depending on the response to treatment. Since Uibarri et al. reported the efficacy of octreotide for treating chyle leakage after laryngeal cancer surgery in 1990 [28], Pan et al. retrospectively compared these two medication doses with diet alone and noted that pharmacotherapy reduced the time to clinical success (4.4 vs. 11.6 days), time to resumption of oral intake (8.8 vs. 16.4 days), and time to drain removal (12.0 vs. 18.6 days) [29]. Etilefrine, which is a sympathomimetic agent with stimulating effects on α- and β-adrenergic receptors, is used as a vasopressor to treat hypotension. In the lymphatic system, its sympathetic α1 action causes the thoracic duct smooth muscle to contract, reducing lymph flow [30]. Guillem et al. administered a continuous etilefrine infusion of 5 mg/h to three patients with chylothorax after surgery for esophageal cancer and reported that all patients responded successfully (median duration of administration: 12 days; median interval to onset of effects: 1 day) [31].

In recent years, there have been an increasing number of reports of treatment for lymphorrhea/chylorrhea using lymphangiography and embolization by IR [8,9]. Lymphangiography was previously performed using the classic method of incising the skin on the dorsum of the foot. Since this method requires technical skills and is invasive, in recent years, intra-inguinal lymph node lymphangiography, which is performed under ultrasound guidance and is less invasive and relatively simple, has become mainstream. Lymphangiography is performed to identify the site of lymphatic damage before surgical intervention for postoperative lymphorrhea. However, because Lipiodol^®^ is viscous and has been found to have a therapeutic effect on lymphorrhea by inducing inflammation at the site of lymphatic damage, it is currently also used to treat post-surgical lymphorrhea [32,33]. The improvement rate of lymphorrhea and chylorrhea by lymphangiography and embolization has been reported as 35–67% in lymphangiography using Lipiodol^®^ alone, and 72–89% by combining Lipiodol^®^ with an embolizing agent [8,9,32]. IR for lymphorrhea is an excellent treatment that is minimally invasive and has a high success rate. In our cases, the success rate of IR treatment was 100%, and there were no adverse events associated with the procedure. However, there are very few radiologists skilled in IR in Japan. Hence, at facilities that cannot perform IR, referral to a facility that can perform IR should be considered.

Surgery for postoperative lymphorrhea and chylorrhea should be performed only in cases refractory to conservative treatment or IR. The problems with surgery are as follows: ① during the first 2 to 6 weeks postoperatively, the intense inflammatory response and tissue fragility make reoperation more difficult, increasing the likelihood of further complications, and ② it is difficult to identify the site of leakage during surgery. Therefore, it is extremely important to decide when to perform reoperation for curing lymphorrhea and chylorrhea. Baniel et al. reported that injection of milk or a fat emulsion into the gastrointestinal tract before or during surgery makes it easier to identify the site of the leak [22]. If the leakage site cannot be identified, nonselective suturing of the tissue around the aorta or spraying of fibrin glue is reportedly effective [6].

**Figure 3:**
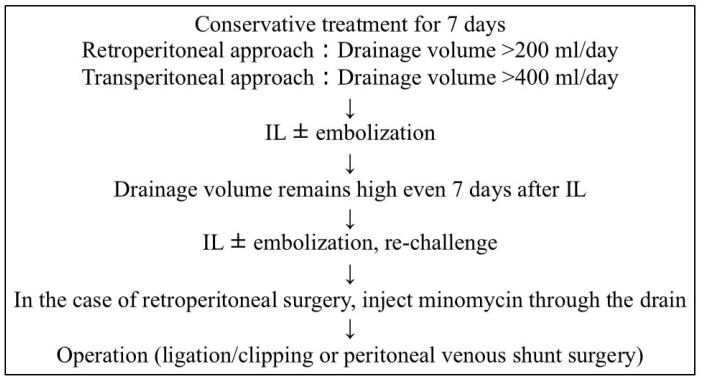
Treatment algorithm for postoperative lymphorrhea and chylorrhea (partial revision of the algorithm proposed by Rose et al. [6]). IL: intranodal lymphangiography.

Rose et al. previously conducted a meta-analysis of 146 articles including 523 patients (urological surgery: 213 patients) who developed iatrogenic chylous ascites after retroperitoneal surgery, and reported that 68.5% of patients improved with conservative treatment [6]. The median treatment period for patients who improved with conservative treatment was 11 days (8–16 days), while the median treatment period in patients who required additional treatment due to the inefficacy of conservative treatment (e.g., lymphangiography or surgical treatment) was 22 days (14–42 days), and the median drainage volume was 750 mL/day (600–1140 mL/day). Additionally, lymphangiographic embolization was successful in curing chyle fistulas in 209 (96.7%) of 216 patients who did not improve with conservative treatment. The median time to improvement of chylous ascites after lymphangiography was 6 days (4–8 range). If the drainage volume remains high 7–10 days following lymphangiography, repeat lymphangiography with embolization is appropriate and has been successful in several reports [34,35]. In the meta-analysis of Rose et al., of the seven patients who did not improve with lymphangiography, six patients underwent surgical treatment, and one patient underwent peritoneovenous shunting, ultimately resulting in a cure in all patients. Based on the data obtained, they generated the first evidence-based treatment algorithm for chylorrhea that encompasses various retroperitoneal dissection procedures across several surgical subspecialties. However, in their algorithm, conservative treatment was performed for 21 days, and the drainage volume was set at 750 mL/day as an indicator for changing treatment, resulting in a longer hospitalization period. Therefore, we revised part of the treatment algorithm developed by Rose et al. In order to shorten the treatment period for lymphorrhea and chylorrhea, we decided to evaluate the effectiveness of conservative treatment within 7 days. The amount of drainage fluid used as a cut-off for intervention was changed to 400 mL/day for surgery performed using a transperitoneal approach and 200 mL/day for retroperitoneal surgery. The retroperitoneal approach is often selected for urological surgery. Since there is no absorption of lymphatic fluid from the peritoneum following the retroperitoneal approach, the timing of treatment intervention is set to reduce the amount of drainage compared to the transperitoneal approach. Additionally, adhesive therapy by the injection of minocycline through the drain before surgical treatment has also been described as a treatment option for lymphorrhea and chylorrhea after surgery via the retroperitoneal approach (Figure 3).

We acknowledge several limitations. The small number of cases and single-center retrospective study design may limit the findings regarding treatment efficacy and treatment-related adverse events. Future investigation should include prospective multicenter studies to comprehensively understand the impact of shorter treatment durations to validate the results of this study.

## 5. Conclusions

The advantage of our proposed treatment algorithm for postoperative lymphorrhea and chylorrhea is that conservative treatment is evaluated in one week, and if the treatment effect is insufficient, IR is performed, thereby shortening of the duration of the hospital stay. Lymphangiography (±embolization) should be implemented in lymphorrhea and chylorrhea patients unsuccessfully managed with conservative measures, and surgical treatment should be performed as a last resort in refractory patients. At institutions where IR cannot be performed, we recommend referral to an institution that has access to IR facilities when conservative treatment does not result in improvement.

## Figures and Tables

**Table 1:** Characteristics of patients with postoperative lymphorrhea or chylorrhea.

	n = 28 Mean ± SD (% or Min–Max)
Age (years)	65.0 ± 9.9 (35–77)
Sex (n)	Male: 26 (92.9%), Female: 2 (7.1%)
Primary tumor location (n)	Bladder: 14 (50.0%), Prostate: 12 (42.9%), Renal pelvis: 2 (7.1%)
Surgical method (n)	Robot: 5 (17.9%), Laparo: 6 (21.4%), Open: 17 (60.7%)
Approach (n)	Transperitoneal: 17 (60.7%), Retroperitoneal: 11 (39.3%)
No. of LNs removed (n)	25.3 ± 15.0 (3–63)
pN (n)	3 (10.7%)
No. of positive LNs (n)	0.9 ± 3.4 (1–17)
Type of leakage (n)	Lymphorrhea: 24 (85.7%), Chylorrhea: 4 (14.3%)
Drainage volume before treatment (mL/day)	373.3 ± 444.7 (40–2300)
Drainage volume after treatment (mL/day)	81.1 ± 128.8 (0–485)
Duration of drain placement (days)	18.3 ± 14.3 (0–69)

LN: lymph node; SD: standard deviation; min: minimum; max: maximum.

**Table 2:** Treatment methods for postoperative lymphorrhea and chylorrhea.

1. Conservative treatment
· Diet therapy (fasting, fat-restricted diet, medium-chain fatty acid diet)
· Drainage + adhesion therapy with minocycline injection
· Octreotide
· Etilefrine
2. Lymphangiography, Embolization
· lipid-soluble iodinated contrast agent
· Embolization with NBCA (N-butyl-2-cyanoacrylate)
3. Surgical treatment
· Ligation and clipping
· Peritoneal venous shunt
· Spraying bioadhesives, etc.

## Data Availability

It is available upon reasonable request.
